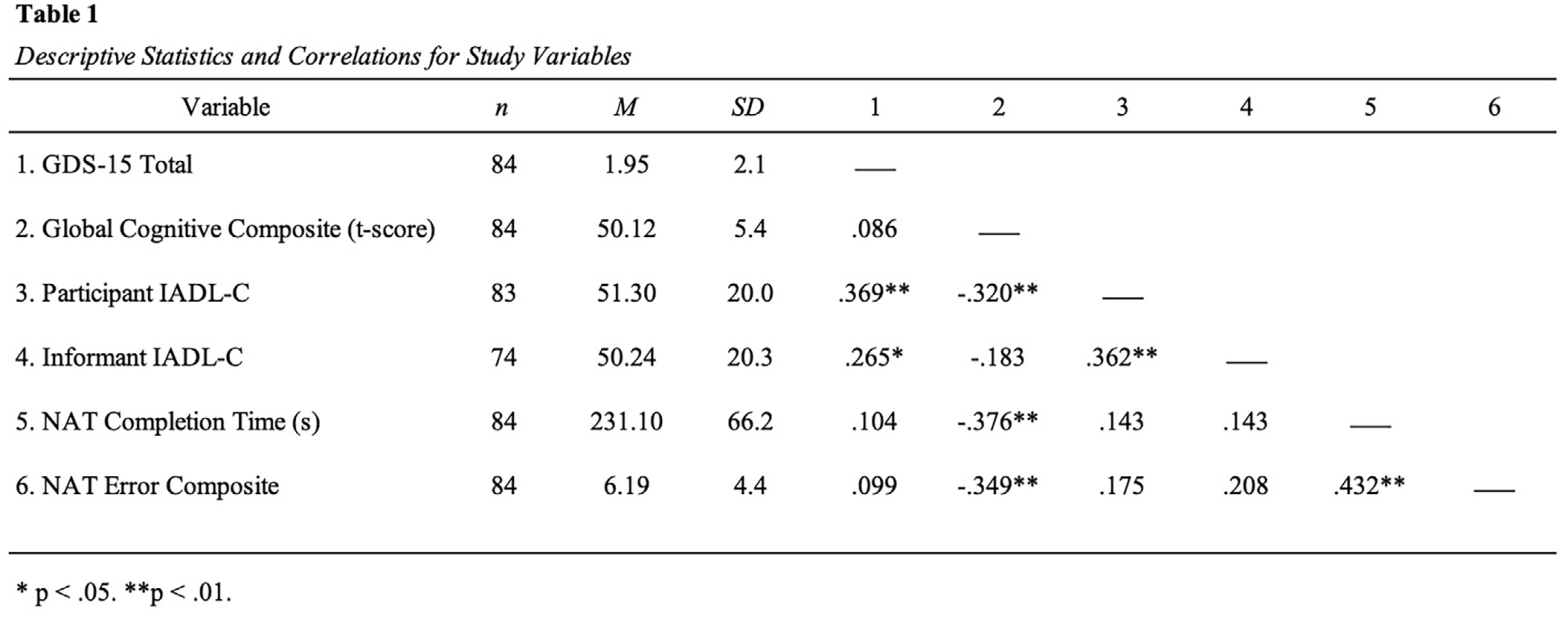# Differential relations between depressive symptoms and everyday function in older adults using multiple modes of functional assessment

**DOI:** 10.1002/alz.093203

**Published:** 2025-01-03

**Authors:** Kimberly Halberstadter, Marina Kaplan, Moira Mckniff, Sophia L. Holmqvist, Molly B. Tassoni, Stephanie M. Simone, Tania Giovannetti

**Affiliations:** ^1^ Temple University, Philadelphia, PA USA

## Abstract

**Background:**

The impact of depressive symptoms on everyday function in older adults remains poorly understood. Depression may decrease motivation, impair cognition, and/or bias self‐reports of functional ability. The present study examined relations between depressive symptoms and everyday function as measured by self‐report, informant‐report, and an objective performance‐based measure which evaluates functional/cognitive capacity but requires only minimal motivation.

**Method:**

88 community dwelling older adults with normal cognition or mild cognitive impairment completed the Geriatric Depression Scale (GDS‐15), tests of cognitive abilities, and the performance‐based Naturalistic Action Test (NAT), which requires preparation of a breakfast and lunch using real objects presented on a table. Video recordings of the NAT were scored for completion time and errors. Participants and their study partners also completed the Instrumental Activities of Daily Living – Compensation (IADL‐C) Questionnaire, which evaluates the ability to perform a range of everyday tasks in daily life. A Spearman correlation matrix was used to assess associations among measures.

**Result:**

GDS‐15 scores were significantly associated with both questionnaires (participant IADL‐C: r = .369, p < .001; informant IADL‐C: r = .265, p = .022) but did not correlate with NAT scores. Global cognitive composite scores were negatively correlated with self‐reported IADL‐C scores (r = ‐.320, p = .003) and NAT scores (completion time: r = ‐.376, p < .001; errors: r = ‐.349, p < .001), but did not correlate with informant‐reported IADL‐C scores. Ancillary analyses showed the two questionnaire measures were significantly correlated (self‐report x informant‐report IADL‐C, r = .362, p = .002) but did not correlate significantly with NAT scores, and the GDS‐15 did not correlate with the cognitive composite.

**Conclusion:**

Among community dwelling older adults, depressive symptoms were associated with participant‐reported and informant‐reported everyday function, but not with performance‐based tasks or cognitive abilities. The pattern of correlates suggests that depressive symptoms may affect function in everyday contexts by decreasing motivation to engage in daily activities without altering cognitive capacity to perform everyday tasks. Future research should investigate relations among specific depressive symptoms, such as apathy, and multiple measures of everyday function.